# Simultaneous stereotactic body radiation therapy of a primary non-small cell lung cancer and synchronous carcinoma in situ in a medically inoperable patient: case report

**DOI:** 10.1186/1748-717X-8-213

**Published:** 2013-09-13

**Authors:** Feras Oskan, Christine Kornhuber, Grit Krause, Dirk Vordermark

**Affiliations:** 1 Department of Radiation Oncology, Martin-Luther University Hospital, Halle-Wittenberg, Dryanderstraße 4, 06110, Halle (Saale), Germany; 2 Department of Radiation Oncology, University Hospital of Saarland, Kirrbergerstraße, 66421, Homburg, Saar, Germany; 3 Department of Pneumono-Oncology, Lung Hospital of Ballenstedt/ Harz gGmbH, Robert-Koch-St.26-27, 06493, Ballenstedt, Germany; 4 Klinik und Poliklinik für Strahlentherapie, Universitätsklinikum Saarland, Kirrbergerstraße 100, 66421, Homburg, Saar, Germany

**Keywords:** Pulmonary carcinoma in situ, Synchronous multiple lung cancer, Roentgenographically occult lung cancer, Stereotactic body radiation therapy, Endoscopic intervention, Radiographic marker

## Abstract

The co-incidence of synchronous intraepithelial neoplasia and early stage invasive lung cancer is not a rare phenomenon. The need for curative treatment and the invasive potential of squamous cell pulmonary carcinoma in situ have been a topic of controversy. Surgical resection still remains the treatment of choice. Varieties of endoscopic techniques such as brachytherapy were developed as an alternative to surgery in selected patients. External beam radiation therapy has been used traditionally in combination with endobronchial brachytherapy in the treatment of roentgenographically occult lung cancer, and can be offered for all patients, but is handicapped, because these tumors are radiographically invisible. We report the first case of a pulmonary carcinoma in situ that was successfully treated with stereotactic body radiation therapy.

## Background

The co-incidence of synchronous intraepithelial neoplasia and early stage lung cancer is not a rare phenomenon. In-situ and early invasive lesions detectable only by bronchoscopy have been referred to as roentgenologically occult lung cancer. A significant prevalence of synchronous roentgenographically occult lung cancer as well as metaplasia and dysplasia was reported in surgical series of patients with resectable roentgenographically visible lung cancer and estimated at 9% [1].

The need for curative treatment and the invasive potential of squamous cell pulmonary carcinoma in situ have been a topic of controversy [2,3]. Without therapeutic interventions, a progression of carcinoma in situ into invasive cancer has been reported to occur in 21% over 4–17 months [4]. Surgical resection still remains the treatment of choice for roentgenographically occult lung cancer providing survival rates of 96% and 94% at 5 and 10 years, respectively [5]. Nevertheless, patients with primary lung cancers in two separate sites, where one site represents roentgenographically occult cancer, and the other site roentgenographically visible primary lung cancer, might be medically inoperable or they refuse surgery. For those patients, a variety of endoscopic techniques could be offered as treatment options [2,3,6,7].

External beam radiation therapy [8] has traditionally been used in combination with endobronchial brachytherapy in the treatment of roentgenographically occult cancer, and been applied alone as an alternative to surgery and endoscopic interventions [9].

We report on a case of synchronous pulmonary carcinoma in situ and roentgenographically visible early-stage primary non-small-cell lung cancer in a medically inoperable patient, where both lesions were treated simultaneously with hypofractionated stereotactic body radiation therapy.

## Case presentation

A 78-year-old Caucasian male patient was admitted to our hospital in February 2011 for evaluation of an incidental left lower lung mass, which was detected on chest X-ray performed due to exacerbation of known GOLD stage IV chronic obstructive pulmonary disease (COPD).

Chest computed tomography (CT) showed a 2.1 × 2.6 cm sized mass in the left S6 segment with contact to the pleura.

Flurdesoxyglucose whole-body positron emission tomography (FDG-PET) demonstrated a pathologic intensive glucose metabolism in the left lower lobe with standardized uptake value (SUV) of 14.1; and much lower metabolism in the subcarinal, left mediastinal and right hilar lymph nodes (SUV=2.7-3.3). No distant metastases were observed. Magnetic resonance imaging of the skull revealed no signs of brain metastasis. Transthoracic fine needle aspiration was performed to confirm the malignancy of the left lower lobe mass. This revealed moderately differentiated, not otherwise specified, non-small cell lung cancer.

Fiberrotic bronchoscopy demonstrated no abnormal finding in the trachea and main bronchi. However, the longitudinal mucosal folds were thickened at the orifice of the right upper lobe bronchus, from which a tissue sample was obtained. The pathology reports indicated a squamous cell carcinoma in situ as well as fragments of squamous dysplasia and metaplasia. To exclude the malignancy in mediastinal and hilary lymph nodes, transoesophageal endosonography was performed and revealed several and enlarged lymph nodes in positions 7 and 4 L (up to 1.6 cm), from which specimens were obtained. Cytologically as well as pathologically there was no indication for malignancy in these lymph nodes. The tumor was classified as cT2a cN0 cM0 with synchronous carcinoma in situ (CIS).

Because of medical co-morbidities, poor performance status, and compromised lunge function, stereotactic body radiation therapy (SBRT) of the left lower lobe mass and cryotherapy of the CIS disease in the right upper main bronchus were recommended as the most appropriate curative option by the multidisciplinary team. In March 2011, the patient underwent a second bronchoscopy to deliver cryotherapy of the known lesions in the orifice of the right upper lobe main bronchus with 3 freeze cycles of 25 seconds each. There was no post-procedural complication. However, during bronchoscopy, a newly manifested thickness of surrounding mucosa of the right segmental bronchus B1 was recognized. To prevent airways obstruction by the necrotic tissue, second bronchoscopy was performed a week later. It demonstrated further regression of the granulation in the floor of right upper lobe bronchus, but a widened bifurcation between the right B1 and B2 bronchi. The pathological reports confirmed the persistence and multifocality of carcinoma in situ.

**Figure 1:**
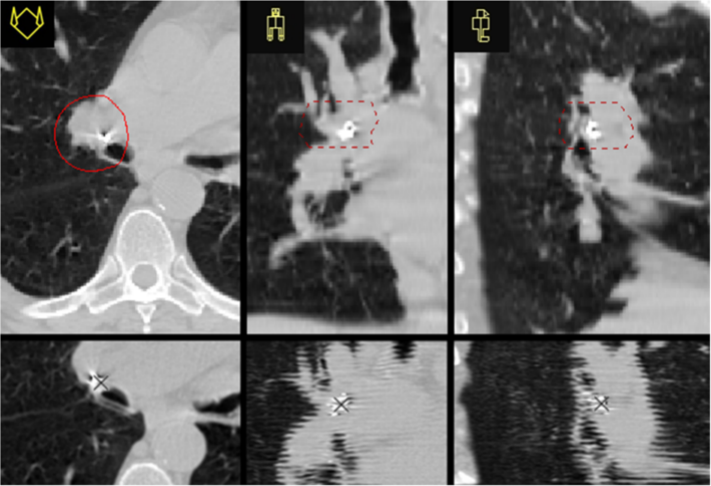
**Treatment planning computer tomography of carcinoma in situ.** Planning CT shows the position of the Visicoil radiographic marker in transversal, sagittal and coronal sections with respect to the right upper lobe main bronchus and demonstrates the ellipsoidal PTV with respect to the Visicoil radiographic marker and the left upper lobe main bronchus in transversal, sagittal and coronal slices. Upper row: fast helical scan of planning CT; lower row: slow helical high resolution scan acquired during verification procedure. Whereas in fast scanning mode almost no motion of radiographic marker can be seen, breathing motion becomes apparent in slow helical scanning mode. For verification of correct treatment position therefore the latter scanning mode was chosen. Radiographic marker movement center was found by means of a 3D-cursor (cross).

After a second discussion of the case by the multidisciplinary team, simultaneous SBRT of the carcinoma in situ was planned at the same time with the SBRT of the left lower lobe mass. Before SBRT treatment planning, the patient underwent bronchoscopy to place a Visicoil (0.5×10 mm) fiducial marker per endobronchial ultrasound-transbronchial aspiration (EBUS-TBNA) near the lymph node station 11 R, since the placement of Visicoil via EBUS in the area of the in situ lesions was technically impossible [Figure 1].

Prior to radiation treatment, a dynamic planning CT with three breathing phases was performed. The internal target volume (ITV) was constructed by union of delineations of the gross tumor volume (GTV) of the left lower lobe mass in all breathing phases, and a margin of 5 mm in all directions was added to create the planning target volume (PTV). A dose of 12.5 Gy × 3 fractions was prescribed to the 65% isodose covering this PTV.

For carcinoma in situ disease there was no GTV to be delineated, thus we pragmatically contoured an ellipsoid as CTV that covered the right upper main bronchus and the segmental bronchi B1/B2 cranially, ventrally and to the right of the Visicoil marker. A margin of 5 mm in all directions to account for the respiratory motion, which was observed on planning CT, was added to create the PTV. A dose of 10 Gy × 3 fractions was prescribed to this PTV (65% isodose).

**Figure 2:**
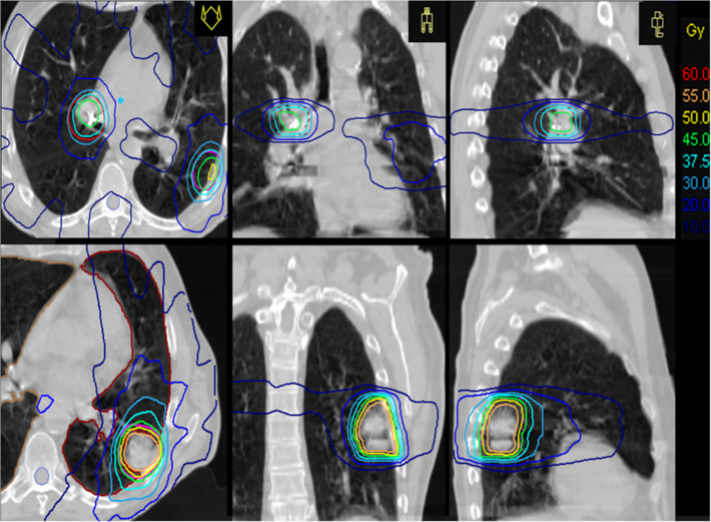
**Radiation treatment planning of both lesions.** Transversal, sagittal and coronal slices representative of SBRT isodose distribution for carcinoma in situ (upper row) and for left lower lobe mass (lower row).

**Figure 3:**
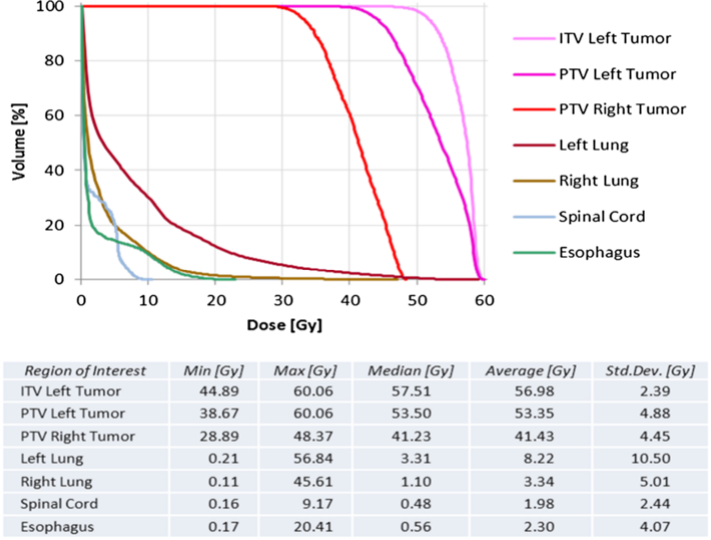
**Dose-constrains for both lesions.** Dose volume histograms showing dosimetrical parameters analyzed including targets and organs at risk.

Multiple static beams with energies of 6 MV were used [Figure 2, Figure 3] and the dose was delivered with daily, CT-based image-guidance as well as MV-portal imaging, using the radiographic marker to verify the treatment position. The total dose was delivered in 10 days, with daily sessions alternating between the two lesions.

At follow up in July 2011, a follow-up CT showed partial remission in the left lower lobe mass, which was then 1.5 cm maximum diameter, and no evidence of pneumonitis. A scar of mucosa in the area of in situ carcinoma, but no signs of new endobronchial manifestations were seen at bronchoscopy. No biopsies were performed. The patient reported no adverse advents of SBRT.

**Figure 4:**
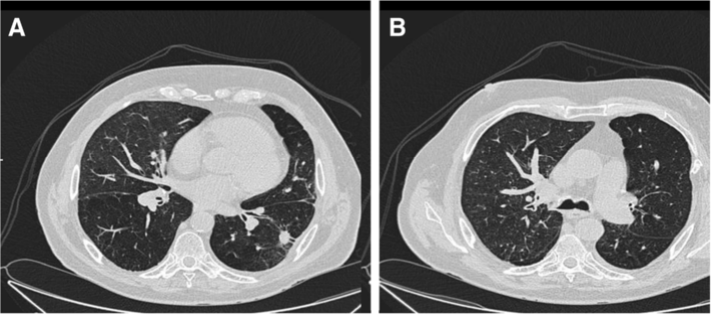
**Follow-up CT after 12 month.** Chest-CT one year after SBRT showing the residual mass in the left lower lobe **(A)**, and hypoventilation changes in the right upper lobe **(B)**.

The patient refused further follow-up. Nonetheless, he was admitted again to the hospital in April 2012 due to exacerbation of COPD. A chest CT [Figure 4] showed no change in size of the left lower lobe tumor and no signs for nodal progression. The patient died in April 2012 due to decompensation of cardiopulmonary function. No endoscopic long-term follow-up data were available.

## Discussion

External beam radiation therapy for carcinoma in situ is also problematic, because these tumors are roentgenographically invisible. It is, therefore, a challenging task for the radiation oncologist to localize clinical target volume (CTV) in space and in time, since there is no gross tumor volume (GTV) be delineated. This inherent uncertainty of CTV must be balanced with the utilization of technological sophistication.

Due to the high efficacy in the treatment of superficial lesions, high-dose brachytherapy could be a definitive treatment for carcinoma in situ or endobronchial occult lung cancer with limited invasion. Endobronchial ultrasonography has been reported to result in a change in management in 36% of tumors thought to be curable by endobronchial therapy after bronchoscopy [10].

A recent large retrospective study [11] of 226 patients, of those 60 patients with carcinoma in situ, treated with high-dose brachytherapy alone showed no difference in local control between patients with in situ carcinomas and those with invasive carcinomas, probably because they were classified as non-invasive on biopsies though infiltrating on bronchoscopy. Furthermore, outcomes were worse for proximally located tumors and in patients treated with one catheter. These data suggest that the application of brachytherapy may be limited by bronchoscopic access, proximal tumor location, multiple bronchoscopy for catheter placement, geographic miss in dosimetric coverage of the target volume when using one catheter, and by post-procedural complications such as fatal hemoptysis or fistula formation.

Compared with conventionally planned radiotherapy, brachytherapy has the advantage of application of high-dose irradiation in tumor with rapid dose reduction outside the treatment volume. This favorable dosimetrical characteristic can be emulated by stereotactic guidance of external beam radiotherapy. The idea of use SBRT to deliver high-dose-rate-like hypofractionated radiotherapy came originally from Molla et al. [12], who suggested a fractionated stereotactic radiotherapy boost for gynecological tumors as an alternative to high-dose brachytherapy and has received some attention in recent years in the treatment of cervical cancer [13]. We suggest that this idea could be potentially adopted in the radiation therapy of roentgenographically occult lung cancer. There is growing evidence, that it is possible to construct SBRT plans that closely emulate high dose brachytherapy dosimetry and deliver the plans non-invasively.

A major issue for the treatment of these lesions with SBRT is, however, that high-dose delivery precision requires a higher degree of accuracy in target volume delineation and in patient set-up during the radiation treatment course. Radiographic markers are well known in the postoperative setting, where there is actually no GTV to be delineated, and are standard tools for image-guided radiotherapy (IGRT) and robotic radiosurgery, and can be used for making invisible lesions “visible” for guiding high-dose SBRT by target volume delineation and positional verification.

In a prospective pilot series conducted by Malfiat et al. [14] to assess the feasibility and safety of EZ-Clips HX 610–090 (Olympus, Aartselaar, Belgium) in inoperable patients with roentgenographically occult lung cancer, one patients underwent high-dose external radiation therapy. Two clips were used to delineate the clinical target volume on the treatment planning system and to verify the correct positioning of the patients during the treatment course. No adverse events were observed. Both clips had disappeared, one was already lost at the time of treatment planning, and the other was lost during radiation treatment.

Anderson et al. [15] reported on the placement of 127 fiducials in 32 patients with mediastinal and centrally located thoracic tumors via flexible bronchoscopy as precursor to robotic radiosurgery. Complications included migration after insertion (one fiducial) and dropping in the airways prior to insertion (24 fiducials). Of those, 18 were removed with biopsy forceps, 2 were suctioned, 3 coughed out, and 2 were not retrieved, but were not seen on post-procedure chest x-ray. Three fiducials in two patients embolized via the pulmonary artery without adverse clinical consequence.

Endoscopic Ultrasound-guided fiducials placement as an alternative method has been described by Charabaty-Pishvian et al. [16]. The Doppler function also verifies that there are no interfering vessels between the tumor and the needle, addressing the problem of post-procedural bleeding and embolization via pulmonary artery. These published data suggest that the implantation of radiographic markers via flexible bronchoscopy or EBUS is safe, has no clinically significant side adverse events, and can be offered for most patients. Furthermore, the movement of radiographic markers in space and time has been even studied, and estimated to be clinically acceptable [17].

Nevertheless, high-dose radiation treatment for centrally located tumors, particularly since the “bronchial exclusion zone” of RTOG 0236 is the main location of roentgenographically occult lung cancer, remains the main issue concerning the toxicity of SBRT. In addition to the fractionation scheme, central location of target lesions also appears to be a predictor of normal tissue toxicity. However, a recent review of 20 studies suggests that safe treatment of central tumors can be achieved with hypofractionated concepts with reduced single dose [18].

## Conclusion

The implantation of radiographic marker near or in the roentgenographically occult lung cancer appears to be safe and would allow precise delivery of hypofractionated SBRT to a target that is now radiologically visible and trackable in space and in time.

## Consent

Written informed consent was obtained from patient’s legal guardian for publication of this Case Report and any accompanying images. A copy of the written consent is available for review by the Editor-in-Chief of this journal.

## Abbreviations

COPD: Chronic obstructive pulmonary disease; CT: Computer tomography; CIS: Carcinoma in situ; PET: Positron emission tomography; SBRT: Stereotactic body radiation therapy; GTV: Gross tumor volume; CTV: Clinical target volume; ITV: Internal target volume; PTV: Planning target volume

## Competing interests

The authors declared that they have no competing interests.

## Author’s contribution

FO participated in the radiation management of the patient, collected patient’s data, reviewed literature and drafted the manuscript. GK contributed the endoscopic interventions components of the manuscript. CK performed the radiation treatment planning, and dosimetrical measurement and calculations for this patients. DV contributed the radiotherapy components of the manuscript and revised the article. All authors performed critical review of the manuscript an approved the final manuscript.
